# Clinical research progress of esketamine in perioperative neuroprotection: from mechanisms to therapeutic neuroprotection

**DOI:** 10.3389/fphar.2026.1960869

**Published:** 2026-09-18

**Authors:** Hua Wang, Ce Zhou, YingDong Qu, Hao Luo

**Affiliations:** Department of Anesthesiology, People’s Hospital of Longhua, ShenZhen, China

**Keywords:** anti-inflammatory effects, esketamine, neuroprotection, NMDA receptors, perioperative

## Abstract

During the perioperative period, factors such as ischemia-reperfusion injury, neuroinflammation, and cognitive dysfunction not only significantly affect postoperative recovery, but also are the main causes of postoperative disability and perioperative death. Esketamine is the R-enantiomer of ketamine, possessing N-methyl-D-aspartate (NMDA) receptor antagonistic activity along with multiple anti-inflammatory, anti-apoptotic, and antioxidant properties. Compared to racemic ketamine, esketamine is more potent, acts more rapidly, causes mild respiratory depression, and induces fewer adverse neuropsychiatric effects, making it a promising agent for perioperative neuroprotection. This review aims to provide a comprehensive overview of esketamine’s neuroprotective mechanisms and clinical applications in the perioperative setting, offering insights into future research directions that may advance therapeutic neuroprotection and improve surgical patient outcomes.

## Introduction

Perioperative brain injury represents a significant clinical challenge, as it is a major contributor to postoperative neurocognitive complications such as perioperative cognitive dysfunction (POCD), postoperative delirium (POD), stroke, and neurological deterioration. Studies have found that the incidence of POCD is 15%–40%, and the incidence of POD is 15%–80% (Tang et al., 2026; Kamar et al., 2025). These adverse outcomes are particularly prevalent among elderly and high-risk patient populations undergoing surgery (Han et al., 2023; Zhao C. et al., 2026). The pathophysiology underlying perioperative brain injury is multifactorial, involving neuroinflammation, oxidative stress, neuronal apoptosis, and synaptic dysfunction. Sleep disturbances prior to surgery have also been identified as exacerbating factors that worsen microglial activation and impair neurotrophic signaling pathways, thereby aggravating postoperative emotional and cognitive impairments (Wen et al., 2024). Moreover, excessive activation of NMDA will increase intracellular calcium influx, leading to various neurological diseases and disorders (Rana et al., 2024). Despite advances in anesthetic and surgical techniques, effective neuroprotective strategies to mitigate perioperative brain injury remain limited, underscoring the urgent need for novel therapeutic agents that can target multiple pathogenic mechanisms.

Esketamine has a potent antagonistic effect on NMDA receptors, and its affinity for NMDA receptors is twice that of R-ketamine (Correia-Melo et al., 2020). Compared with ketamine, Esketamine has a stronger anesthetic and analgesic effect, and the incidence of mental side effects is significantly lower (Zhao C. et al., 2026). Moreover, it can regulate the neuroinflammatory response and neurotrophic signaling pathways related to perioperative brain injury (Li et al., 2022). Additionally, esketamine has demonstrated rapid antidepressant effects and the ability to improve postoperative emotional and cognitive outcomes, particularly in aged animal models with preoperative sleep disturbances (Wen et al., 2024; Wang et al., 2026). Beyond its direct central nervous system effects, emerging research has revealed that esketamine may exert indirect neuroprotective actions through modulation of the gut-brain axis (Zhao X. et al., 2026).

In recent years, an increasing amount of evidence has shown that esketamine has neuroprotective effects in various central nervous system diseases, including improving the incidence of postoperative behavioral disorders in children, as well as providing effective sedation and analgesia. This review aims to systematically synthesize the current clinical and mechanistic evidence regarding esketamine’s neuroprotective effects in the perioperative period, thereby providing a theoretical foundation for optimizing neuroprotection strategies and improving postoperative neurological outcomes (Figure 1).

**FIGURE 1:**
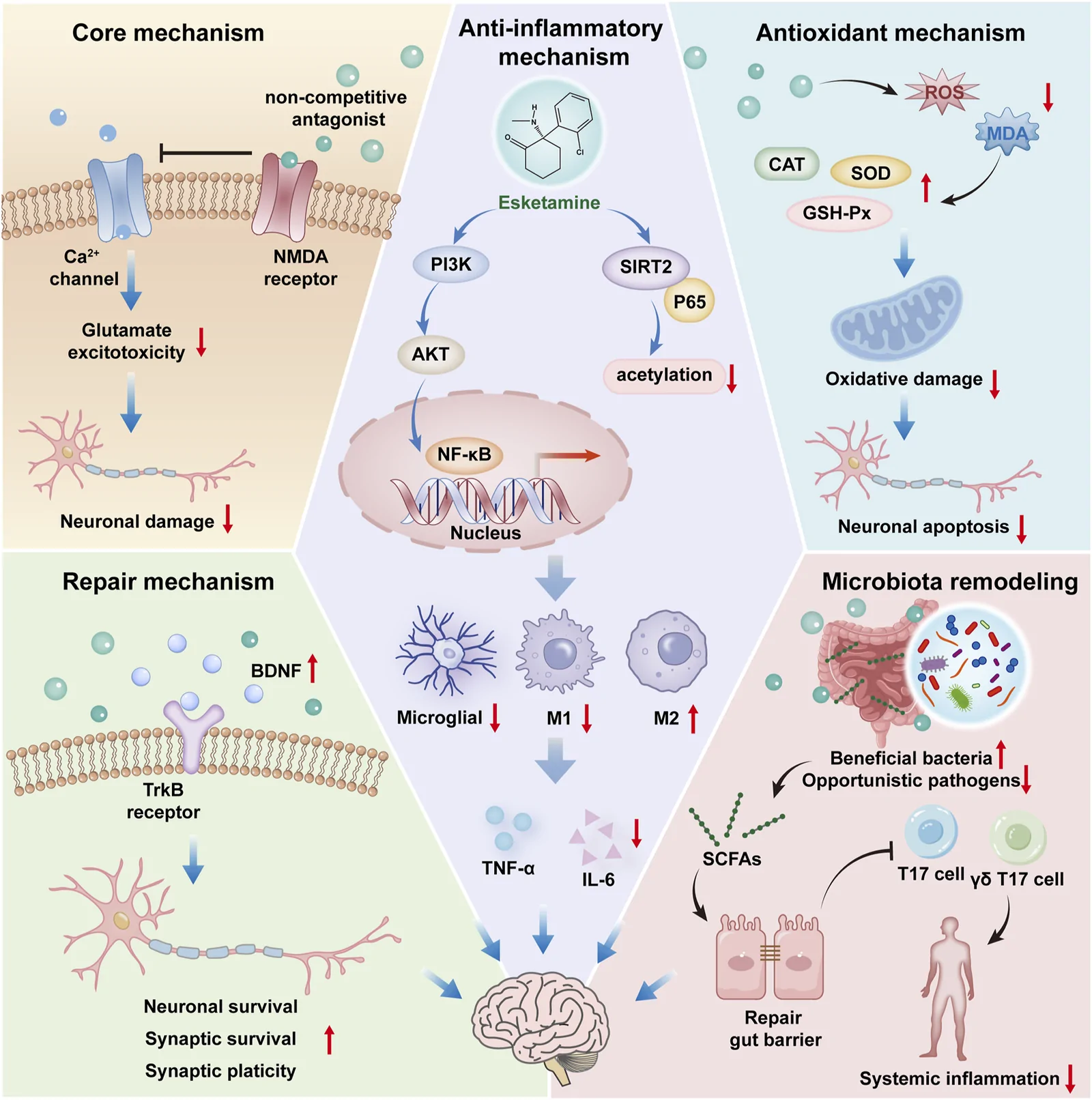
Mechanism diagram of esketamine’s effect on perioperative brain protection. Core mechanism: Esketamine functions as a non-competitive antagonist of the NMDA receptor, thereby blocking calcium (Ca^2+^) influx through the Ca^2+^ channel. This inhibition attenuates glutamate excitotoxicity, ultimately reducing neuronal damage. Anti-inflammatory mechanism: Esketamine suppresses neuroinflammation through two convergent pathways. First, it activates the Phosphatidylinositol 3-Kinase (PI3K)/Protein Kinase B (AKT) signaling cascade, which inhibits the transcriptional activity of nuclear factor-kappaB (NF-κB) in the nucleus. Second, esketamine upregulates SIRT2 expression, promoting deacetylation of the p65 subunit of NF-κB. The combined suppression of NF-κB signaling reduces microglial activation, shifts microglial polarization from the pro-inflammatory M1 phenotype (M1↓) toward the anti-inflammatory M2 phenotype (M2↑), and downregulates the production of pro-inflammatory cytokines including TNF-α and IL-6. Antioxidant mechanism: Esketamine enhances the production of reactive oxygen species (ROS) and malondialdehyde (MDA), while enhancing the activity of endogenous antioxidant enzymes, including catalase (CAT), superoxide dismutase (SOD), and glutathione peroxidase (GSH-Px), thereby alleviating oxidative damage to mitochondria and decreasing neuronal apoptosis. Repair mechanism: Esketamine increases the expression of brain-derived neurotrophic factor (BDNF), which binds to its cognate receptor TrkB. This BDNF/TrkB signaling promotes neuronal survival, enhances synaptic survival, and improves synaptic plasticity, facilitating functional recovery of damaged neural circuits. Microbiota remodeling: Esketamine modulates gut microbiota composition by increasing beneficial bacteria and decreasing opportunistic pathogens. This remodeling promotes the production of short-chain fatty acids (SCFAs), which help repair the integrity of the gut barrier. Additionally, the altered microbiota influences T helper 17 (Th17) cells and γδ T17 cells, ultimately reducing systemic inflammation. The anti-inflammatory, antioxidant, repair, and microbiota-mediated effects collectively converge to protect the brain from injury.

## Pharmacological characteristics of esketamine and its perioperative application basis

### Molecular structure and mechanism of action

Esketamine is the S-enantiomer of racemic ketamine, and its pharmacological properties differ due to its stereochemical structure. Esketamine acts as a non-competitive antagonist at the glutamatergic NMDA receptors, inhibiting excessive excitatory neurotransmission by reducing presynaptic glutamate release and postsynaptic excitotoxicity. This blockade mitigates neuronal overactivation and consequent cell damage, which is particularly relevant in perioperative neuroprotection (Zhou et al., 2023). Beyond NMDA receptors antagonism, esketamine activates intracellular signaling cascades involving phosphorylation of extracellular signal-regulated kinase (ERK) and cAMP response element-binding protein (CREB), which promote the expression of brain-derived neurotrophic factor (BDNF). The upregulation of BDNF enhances synaptic plasticity and facilitates neuronal repair mechanisms, contributing to its neuroprotective and antidepressant properties (Wen et al., 2024). Furthermore, esketamine interacts with multiple targets, including the α-amino-3-hydroxy-5-methyl-4-isoxazolepropionic acid (AMPA) receptor, opioid receptors, monoamine systems, and ion channels. These various mechanisms enable esketamine to rapidly regulate the neural circuits related to pain, emotional regulation, and neuroprotection (Palucha-Poniewiera, 2025).

### Pharmacokinetic characteristics

Compared with ketamine, esketamine has higher lipid solubility and water solubility, and can rapidly pass through the blood-brain barrier and take effect quickly. After intravenous administration of 0.5 mg kg^−1^ esketamine, the time to reach peak plasma concentration (Tmax) is only 70 s (Wang et al., 2026; Wang et al., 2019). After a single dose, esketamine achieves high cerebrospinal fluid concentrations; however, its duration of action is relatively short, favoring its use in single or brief perioperative applications (Tan et al., 2022). The drug undergoes extensive hepatic metabolism primarily via cytochrome P450 enzymes CYP3A4 and CYP2B6, producing metabolites such as S-norketamine and S-hydroxynorketamine. Most of these metabolites are excreted via the kidneys or bile, and subsequently conjugated with glucuronic acid (Wang et al., 2019; Dinis-Oliveira, 2017). Esketamine’s elimination half-life ranges from 2 to 3 h, and it lacks active metabolites with prolonged accumulation, minimizing the risk of drug accumulation with repeated dosing (Wang et al., 2026).

However, pharmacokinetic parameters can be influenced by patient-specific factors including age, hepatic and renal function, and concomitant medications that modulate CYP enzyme activity, necessitating individualized dosing adjustments during the perioperative period (Langmia et al., 2022). Alternative administration routes such as intranasal and oral have been explored, though oral bioavailability is limited due to significant first-pass metabolism, resulting in altered metabolite profiles and reduced central nervous system effects compared to intravenous administration. These pharmacokinetic features underscore esketamine’s suitability for controlled perioperative use, allowing rapid titration and minimizing prolonged sedation or adverse effects.

### Safety and management of adverse effects

In randomized controlled trials involving adult surgical patients, esketamine demonstrated fewer and milder adverse mental events compared to racemic ketamine. Moreover, by carefully adjusting the dosage during administration and conducting monitoring, the incidence of psychotic-like side effects can be further reduced (Kritzer et al., 2022; Wang et al., 2021). Esketamine primarily exerts sympathomimetic effects by inhibiting the reuptake of norepinephrine and epinephrine, thereby increasing circulating catecholamines and elevating heart rate and blood pressure. Additionally, population-based studies indicate a slightly higher risk of cardiac arrest associated with esketamine compared to racemic ketamine and oral antidepressants, warranting cautious use in patients with pre-existing cardiovascular disease such as coronary artery disease and uncontrolled hypertension (Lee et al., 2026). The risk of nausea and vomiting is relatively low, with reported incidence rates of 10%–15%, which is less than that observed with many other anesthetic agents. Prophylactic and therapeutic use of antiemetics effectively manages these symptoms in the postoperative period (Qi et al., 2024). Gender differences have also been observed in adverse drug reactions: men are more prone to hypertension and urinary retention, while women are more likely to experience suicidal ideation and elevated liver enzymes, highlighting the importance of individualized risk assessment (Yang and Chen, 2024). The pharmacological effects of esketamine include increased cerebral blood flow and intracranial pressure (ICP), which require careful consideration in patients with intracranial space-occupying lesions, traumatic brain injury, or cerebral edema. In such cases, a thorough assessment of the risk-benefit ratio is essential, and adjunctive measures such as hyperventilation and concomitant intravenous anesthesia may be employed to mitigate elevated ICP.

However, esketamine is subject to non-medical abuse. Long-term misuse can lead to urinary tract toxicity, as high concentrations of esketamine and its metabolite, norketamine, are excreted in urine. Direct contact with the bladder urothelium causes significant toxicity, clinically manifesting as severe frequency, urgency, difficulty urinating, and gross hematuria (Morgan et al., 2012). Moreover, esketamine abuse often induces dissociative symptoms and persistent cognitive impairments, such as cognitive decline and social withdrawal. The severity of these cognitive deficits is strongly dose-dependent, and female users appear more susceptible to dependence, which may be related to gender differences in pharmacokinetics and neurobiology (Vasudeva et al., 2026; Le et al., 2022; Levinstein et al., 2026).

Overall, long-term safety data remain limited, particularly regarding potential neurocognitive effects, urotoxicity, and abuse liability. Ongoing pharmacovigilance, well-designed clinical trials, and careful patient selection are crucial for clarifying these risks.

## Research on neuroprotective mechanisms of esketamine

### Direct inhibition of neuronal apoptosis and excitotoxicity

NMDA receptors are key mediators of excitotoxic neuronal injury during cerebral ischemia-reperfusion (Tang et al., 2023). In the adult rat model of cerebral ischemia-reperfusion, excessive activation of NMDA receptors leads to pathological calcium influx, triggering downstream cascades including reactive oxygen species (ROS) generation and mitochondrial dysfunction, which culminate in neuronal apoptosis (Alamoudi, 2026; Gao et al., 2025). Esketamine’s blockade of NMDA receptors effectively reduces calcium overload, thereby attenuating oxidative stress and preserving mitochondrial integrity. Moreover, this study that administration of esketamine significantly decreases infarct volume by 30%–45%, indicating substantial neuroprotection against ischemic injury (Gao et al., 2025). Notably, esketamine preserves pyramidal neurons in the hippocampal CA1 region, a brain area highly vulnerable to ischemic damage, by preventing necrosis and apoptosis. The timing of esketamine administration is critical; its neuroprotective efficacy is most pronounced when given during the early reperfusion phase within 0–3 h post-ischemia, highlighting a therapeutic time window advantageous for perioperative brain protection (Kawczak et al., 2024). This early intervention likely interrupts the excitotoxic cascade before irreversible neuronal damage ensues. These findings underscore esketamine’s capacity to directly inhibit neuronal apoptosis and excitotoxicity through NMDA receptors antagonism, positioning it as a promising agent for perioperative neuroprotection in ischemic brain injury contexts. However, in addition to NMDA receptor antagonism, esketamine interacts with multiple receptor systems, the roles of which in promoting neuroprotective effects are currently under active investigation.

### Modulation of glial cell-mediated neuroinflammatory responses

Beyond direct neuronal effects, esketamine modulates neuroinflammation by regulating glial cell activity. Activation of microglia and astrocytes leads to the release of pro-inflammatory cytokines such as interleukin-1β (IL-1β) and tumor necrosis factor-alpha (TNF-α), exacerbating neuronal damage and blood-brain barrier (BBB) disruption (Zhu et al., 2023; Han et al., 2026). Esketamine has been shown to suppress this glial activation and the associated inflammatory milieu. Moreover, compared with R-ketamine, esketamine shows a more significant anti-inflammatory effect. In a study using a mouse model of depression induced by lipopolysaccharide (LPS), esketamine significantly alleviated depressive-like behaviors, reduced the levels of pro-inflammatory factors in the medial prefrontal cortex (mPFC), and enhanced the interaction between SIRT2 and the nuclear factor kappa B (NF-κB) subunit p65, decreasing its acetylation and inhibiting the expression of pro-inflammatory genes. These effects were not observed in R-ketamine (Lin et al., 2025). Mechanistically, esketamine activates the phosphatidylinositol 3-kinase (PI3K)/Protein Kinase B (Akt) signaling pathway and inhibits NF-κB signaling, which collectively reduce microglial polarization toward the pro-inflammatory M1 phenotype while promoting the anti-inflammatory M2 phenotype (Tian et al., 2025). This phenotypic shift diminishes the secretion of deleterious cytokines and fosters a neuroprotective environment. Concurrently, esketamine decreases astrocytic release of interleukin-6 (IL-6) and matrix metalloproteinase-9 (MMP-9), key mediators implicated in BBB breakdown and secondary neuronal injury. By preserving BBB integrity, esketamine mitigates infiltration of peripheral immune cells and edema formation, further protecting neural tissue (Ju et al., 2025). Clinical biomarker studies in surgical patients receiving neuroprotective anesthesia have observed reductions in MMP-9 and S-100 protein levels, consistent with attenuated neuroinflammation and glial activation. These anti-inflammatory effects of esketamine contribute to improved cognitive outcomes and reduced postoperative delirium incidence, supporting its role in modulating glial-mediated neuroinflammation during the perioperative period (Zhu et al., 2023).

### Promotion of neurotrophic factor expression and synaptic repair

Esketamine also facilitates neuroprotection by enhancing neurotrophic support and synaptic plasticity, essential for neuronal survival and functional recovery after ischemic insults. BDNF and its receptor tropomyosin receptor kinase B (TrkB) are central to neuronal survival, synaptogenesis, and synaptic plasticity (Huang et al., 2025). Esketamine administration upregulates BDNF expression, particularly in the ischemic penumbra, the region surrounding the infarct core that retains salvageable tissue. However, in the depression model of mice, R-ketamine can induce sustained antidepressant effects by increasing the BDNF-TrkB signal and synaptic formation in the prefrontal cortex (PFC), CA3 and dentate gyrus (DG), and it shows stronger and longer-lasting antidepressant effects compared to esketamine (Yang et al., 2015). Animal models reveal that a single dose of esketamine elevates hippocampal BDNF levels by two to threefold within 48 h post-treatment, coinciding with restoration of synaptic proteins such as postsynaptic density protein 95 (PSD-95) and synapsin I (Tian et al., 2025). These synaptic proteins are critical for maintaining synaptic structure and neurotransmitter release, respectively, indicating enhanced synaptic repair and connectivity. The sustained increase in neurotrophic signaling and synaptic protein expression supports recovery of cognitive functions impaired by perioperative ischemia. Moreover, esketamine’s ability to promote synaptic remodeling may counteract perioperative cognitive dysfunction by restoring neural network integrity. This neurotrophic and synaptic repair mechanism complements esketamine’s anti-apoptotic and anti-inflammatory actions, collectively contributing to its comprehensive neuroprotective profile in the perioperative setting (Zhang L. M. et al., 2023).

## Role of gut microbiota in esketamine-mediated neuroprotection: gut-brain axis mechanisms

### Indirect regulation of perioperative brain injury by gut microbiota

Perioperative stress is a critical factor that disrupts the balance of the intestinal microbiota, leading to dysbiosis and compromising the integrity of the BBB, thereby increasing the risk of perioperative brain injury, including cognitive dysfunction and delirium. Emerging evidence from animal models demonstrates that anesthesia and surgery lead to the reduction of beneficial bacteria such as Ruminococcus and Bifidobacterium, and an increase in opportunistic pathogens including *Enterococcus* and *Staphylococcus* species. This microbial imbalance has profound implications for brain health during the perioperative period. The depletion of beneficial microbes compromises the production of short-chain fatty acids (SCFAs), particularly butyrate, which plays a pivotal role in maintaining the integrity of the BBB (Luo et al., 2021), and esketamine can restore the damaged intestinal microbial diversity and metabolic function due to surgical stress. By rebalancing the intestinal microbiota, it indirectly enhances the integrity of the blood-brain barrier and reduces the neuroinflammatory response, thereby exerting neuroprotective effects during perioperative periods (Zhao X. et al., 2026; Gong et al., 2025).

Moreover, perioperative stress triggers the expansion of pro-inflammatory immune cell subsets within the mouse gut, notably γδ T17 cells and Th17 cells, which produce the potent cytokine interleukin-17A (IL-17A). These cells can migrate from the intestinal mucosa to the CNS, where they contribute to neuroinflammatory injury by promoting microglial activation and neuronal damage (Handke et al., 2021; Xu et al., 2025). Esketamine has been shown to reduce the activation of γδ T17 and Th17 cells within the gut and inhibit their trafficking to brain tissue, resulting in decreased IL-17A levels in the CNS (Zhao X. et al., 2026). This immunomodulatory effect is independent of esketamine’s direct antagonism of central NMDA receptors, indicating that esketamine exerts neuroprotection via a novel gut-immune-brain axis pathway. By attenuating the gut-derived immune cell infiltration and subsequent neuroinflammation, esketamine mitigates perioperative brain injury and cognitive decline. This mechanism highlights the importance of peripheral immune regulation in esketamine’s neuroprotective profile and suggests that targeting gut-immune interactions may represent a promising therapeutic strategy for perioperative neuroprotection.

### Mechanisms of esketamine-induced gut microbiota remodeling

Repeated administration of ketamine enantiomers, including esketamine, has been shown to reverse stress-induced alterations in gut microbial composition by increasing the abundance of beneficial genera such as *Lactobacillus* and Bifidobacterium while suppressing colonization by pathogenic bacteria (Gong et al., 2025). This microbial remodeling is accompanied by upregulation of intestinal barrier proteins, including Claudin-1 and Occludin, which are essential components of tight junctions that regulate intestinal permeability. The enhancement of barrier function reduces translocation of gut-derived endotoxins into systemic circulation, thereby diminishing systemic inflammatory burden. Restoration of SCFA levels—specifically acetate, propionate, and butyrate—further contributes to neuroprotection by activating G-protein-coupled receptors GPR41 and GPR43 on microglial cells (Szrejder and Piwkowska, 2025). Activation of these receptors inhibits microglial overactivation, a key driver of neuroinflammation. Studies demonstrating that esketamine’s modulation of gut microbiota and intestinal barrier integrity plays a critical role in its neuroprotective efficacy, suggesting that the gut microbiome is a vital mediator of esketamine’s therapeutic actions in the perioperative setting (Zhao X. et al., 2026; Liu et al., 2025).

Although several studies have reported associations between esketamine use and changes in the gut microbiome, these microbial alterations may stem from esketamine’s primary neurochemical and anti-inflammatory effects rather than its initial therapeutic intent. Moreover, studies have shown that high-dose ketamine exposure may impair intestinal homeostasis, disrupt the gut microbiota, compromise intestinal barrier integrity, lead to hippocampal dysfunction, and subsequently result in depressive, anxiety-like, and cognitive deficits (Xie et al., 2024). Therefore, esketamine requires a more precise therapeutic dose to dissect the central effects mediated by the gut.

## Experimental research progress of esketamine in perioperative brain protection

### Effects of esketamine in cerebral ischemia-reperfusion rodent models

In a mouse model of post-stroke chronic stress, particularly middle cerebral artery occlusion (MCAO), esketamine has demonstrated significant neuroprotective effects when administered intravenously at doses ranging from 10 mg/kg as a single bolus. These doses have been shown to improve neurological function scores, indicating both structural and functional brain protection (Zhang L. M. et al., 2023). Histological analyses reveal that esketamine preferentially protects cortical and hippocampal tissues, regions known for their high density of NMDA receptors. This regional selectivity aligns with the drug’s mechanism of modulating excitotoxicity mediated by excessive glutamate release during ischemia-reperfusion injury. The neuroprotection is likely mediated through attenuation of excitotoxic neuronal death, reduction of oxidative stress, and suppression of neuroinflammatory cascades triggered by ischemic insult. These findings underscore esketamine’s potential as a timely intervention to mitigate ischemic brain injury in the perioperative setting (Zhao C. et al., 2026; Wang L. et al., 2025; Wang C. M. et al., 2024).

### Cognitive protection of esketamine in cerebral ischemia-reperfusion rodent models

In mouse models of global cerebral ischemia, such as those mimicking cardiac arrest-induced brain hypoxia, esketamine has shown robust cognitive protective effects, particularly within the hippocampal CA1 region, which is highly vulnerable to hypoxic injury. Administration of esketamine significantly reduces neuronal loss in this area and improves performance in spatial learning and memory tasks, including the Morris water maze, indicating preservation of hippocampal-dependent cognitive functions (Gao et al., 2025). Mechanistically, esketamine modulates the glutamate-glutamine cycle, a critical pathway for maintaining synaptic transmission and neurotransmitter homeostasis after global hypoxia. By restoring balance in this cycle, esketamine alleviates synaptic transmission deficits and preserves the integrity of synaptic signaling networks. Notably, low doses of esketamine have been sufficient to elicit measurable cognitive benefits, surpassing those observed with racemic ketamine, suggesting a dose-dependent and enantiomer-specific neuroprotective profile (Zhang L. M. et al., 2023). This enhanced efficacy at subanesthetic doses may relate to esketamine’s higher affinity for NMDA receptors and its ability to modulate neuroinflammation and oxidative stress more effectively. These findings support the therapeutic potential of esketamine for preventing cognitive decline following global cerebral ischemia and hypoxia, conditions commonly encountered in perioperative cardiac arrest or severe systemic hypoperfusion.

### Effect of esketamine on perioperative cognitive dysfunction (POCD) in rodent models

POCD in mice is typically modeled by combining inhalational anesthetics such as isoflurane or sevoflurane with surgical interventions like splenectomy or exploratory laparotomy. This dual-stressor approach effectively simulates the clinical scenario where patients are exposed to both surgical trauma and anesthesia, which synergistically contribute to cognitive impairment (Li et al., 2022). To comprehensively assess the cognitive domains affected, a battery of behavioral tests is employed. The Morris water maze (MWM) is widely used to evaluate spatial learning and memory, capitalizing on the hippocampus-dependent navigation abilities of rodents. The novel object recognition (NOR) test assesses recognition memory by measuring the preference for novel versus familiar objects, thus evaluating recognition memory circuits. Additionally, contextual fear conditioning (CFC) tests hippocampus-dependent associative memory by measuring freezing behavior in response to a context previously paired with an aversive stimulus. The combination of these tests allows for a multidimensional evaluation of cognitive function, capturing deficits in spatial, recognition, and associative memory domains. Studies have shown that esketamine can effectively alleviate the spatial learning and memory deficits in mice with postoperative cognitive dysfunction (Wen et al., 2024; Niu et al., 2022; Qi et al., 2026). Esketamine also improves synaptic plasticity by reversing surgery-induced inhibition of hippocampal long-term potentiation (LTP), thereby restoring synaptic transmission efficiency to baseline levels. These synaptic benefits are associated with increased BDNF expression in the CA1 region and decreased nuclear translocation of the pro-inflammatory transcription factor NF-κB/p65, indicating a molecular mechanism involving enhanced neurotrophic support and dampened inflammatory signaling (Ren et al., 2022). Importantly, the memory enhancement effects of esketamine persist for at least 7 days post-surgery, suggesting sustained neuroprotection beyond the immediate perioperative period. These findings provide mechanistic insights into esketamine’s ability to preserve cognitive function in the perioperative setting, highlighting its role in modulating oxidative stress, neuroinflammation, and synaptic plasticity pathways that are critical for memory and learning (Li et al., 2025; Li et al., 2024; Wang Y. et al., 2024).

## Effects of esketmaine combined with non-anesthetics drugs in animal and human

### Synergistic anti-inflammatory effects of esketamine combined with lidocaine

The combination of esketamine and lidocaine presents a promising synergistic approach to perioperative neuroprotection by targeting distinct yet complementary inflammatory pathways. Lidocaine, a well-known local anesthetic, exerts anti-inflammatory effects primarily through the inhibition of voltage-gated sodium channels and suppression of pro-inflammatory cytokines such as TNF-α. When used together, lidocaine and esketamine can act on different nodes within the inflammatory cascade, thereby amplifying the overall anti-inflammatory response during the perioperative period (Hu et al., 2024).

Clinical studies have demonstrated that the co-administration of low-dose esketamine with lidocaine effectively maintains postoperative serum levels of IL-6 and C-reactive protein (CRP) below normal thresholds, indicating a significant attenuation of systemic inflammatory responses. By suppressing these markers, the combination therapy may reduce the risk of perioperative neuroinflammation and associated brain injury (Zhu et al., 2025).

Mechanistically, lidocaine’s blockade of voltage-gated sodium channels inhibits the propagation of nociceptive signals and dampens the activation of immune cells that release pro-inflammatory cytokines. Concurrently, esketamine alleviates excitotoxicity by antagonizing NMDA receptors and modulates microglial activation, thereby further suppressing neuroinflammation (Ray et al., 2022).

### Enhanced neuroprotection by combining esketamine with dexmedetomidine

Dexmedetomidine, a highly selective α2-adrenergic receptor agonist, offers neuroprotective benefits through its sympatholytic and antioxidative properties.

Preclinical animal studies have revealed that the combined administration of esketamine and dexmedetomidine significantly decreased the neuronal hyperactivities and the overactive behaviors (Chu et al., 2021).

The complementary pharmacodynamics of esketamine and dexmedetomidine underpin their synergistic neuroprotective effects. Specifically, the unique yet complementary targets of these two drugs jointly establish a synergistic framework that enhances neuroprotection, thereby preserving neural integrity during perioperative stress or neurological injury. Furthermore, esketamine suppresses the production of peripheral pro-inflammatory cytokines, while dexmedetomidine modulates central neuroinflammation by regulating microglial activation. This dual anti-inflammatory network—peripheral and central—works in concert to protect neural tissue from inflammatory damage (Laaksonen et al., 2018; Nummela et al., 2022). This combination therapy also holds promise for optimizing sedation-analgesia protocols in elderly patients undergoing lower extremity fracture surgery, where maintaining physiological stability and minimizing cognitive impairment are paramount (Guo et al., 2026). Moreover, this sedation protocol improves patient comfort and cooperation during spinal anesthesia positioning, with a low incidence of adverse events (Guo et al., 2026).

In conclusion, the co-administration of esketamine and dexmedetomidine offers a robust neuroprotective strategy by integrating NMDA receptors antagonism with α2-adrenergic receptor-mediated sympatholysis and antioxidation. This synergy enhances hippocampal neuron survival and cognitive outcomes in the perioperative setting without exacerbating cardiovascular side effects, supporting its clinical utility for brain protection.

## Comparative analysis of esketamine and arketamine in neuroprotection

### Affinity and selectivity for NMDA receptors

Esketamine (S-ketamine) and arketamine (R-ketamine) are enantiomers of ketamine with distinct pharmacological profiles, particularly regarding their interaction with the NMDA receptor. Esketamine exhibits a significantly higher affinity for NMDA receptors. This higher binding affinity translates into esketamine’s stronger analgesic, anesthetic, and psychotropic effects, making it more potent in clinical anesthesia and pain management settings (Kawczak et al., 2024). Despite this, when it comes to neuroprotection, especially in the context of ischemic neuronal injury, the difference in NMDA receptors affinity does not straightforwardly predict superior efficacy. Both enantiomers appear to offer comparable direct neuroprotective effects on post-ischemic neurons, suggesting that mechanisms beyond simple NMDA receptors antagonism are at play (Wei et al., 2022). This observation aligns with emerging evidence that ketamine’s neuroprotective actions involve modulation of non-NMDA pathways, including BDNF signaling and anti-inflammatory processes. For instance, arketamine’s engagement of microglial ERK-NRBP1-CREB-BDNF pathways contributes to sustained neuroprotection and antidepressant effects, independent of its lower NMDA receptors affinity (Yao et al., 2022). Therefore, the differential receptor binding profiles underscore that neuroprotection by ketamine enantiomers is multifactorial, with receptor binding affinity being only one component.

### Isomer-specific effects on gut microbiota and immune modulation

The gut microbiota and its interaction with the immune system have emerged as critical modulators of neuroinflammation and brain health, influencing outcomes in perioperative neuroprotection. Esketamine and arketamine differ in their capacity to modulate gut microbial communities and subsequent immune responses. Arketamine has been shown to induce a more durable increase in beneficial gut bacteria such as *Lactobacillus* and Bifidobacterium species, with microbiota remodeling effects persisting for over 10 days post-treatment, surpassing the relatively transient 3–5 days effect observed with esketamine (Liu et al., 2025). This prolonged microbiota modulation by arketamine correlates with a more stable reduction in the migration of pro-inflammatory γδ T17 cells from the gut to the brain, a key mechanism implicated in neuroinflammation and neuronal injury. The enhanced metabolic stability of arketamine may underlie these sustained immunomodulatory effects (Zhao X. et al., 2026). Conversely, esketamine’s impact on gut microbiota appears less enduring, which may limit its long-term neuroprotective potential in conditions requiring prolonged intervention. These findings suggest that arketamine’s superior gut microbiota and immune regulatory profile could confer advantages in clinical scenarios involving repeated surgeries or chronic neuroinflammatory states. However, clinical data remain limited, and further studies are needed to validate these preclinical observations and to elucidate the mechanistic pathways linking gut microbiota alterations to central neuroprotection by ketamine enantiomers.

## Clinical application and challenges of esketamine for perioperative care

### Clinical evidence of improved neurocognitve outcomes with esketamine

Current clinical evidence on the perioperative neuroprotective effects of esketamine primarily derives from multiple small-scale randomized controlled trials (RCTs) that have investigated its intravenous administration at doses ranging from 0.25 to 0.5 mg/kg. These studies consistently demonstrate that perioperative intravenous esketamine can reduce the incidence of POD by approximately 30% in patients undergoing cardiac surgery, alongside a reduction in intensive care unit (ICU) length of stay. Several meta-analyses have shown that intravenous ketamine administration in adult patients undergoing general anesthesia not only helps prevent POD and POCD, but also reduces intraoperative opioid consumption, alleviates postoperative pain intensity, without increasing the incidence of PONV (Lin et al., 2024; Zhang et al., 2024; Hung et al., 2025). Esketamine improves cognitive function, with particularly notable effects in elderly and pediatric populations. For example, a retrospective cohort study involving elderly patients undergoing thoracic surgery reported a significant decrease in POD incidence at 7 days postoperatively from 26.7% in controls to 12.0% in the esketamine group, with concomitant improvements in POCD assessed by the Montreal Cognitive Assessment (MoCA) at 1 and 6 months (Wang Y. et al., 2024). In modified radical mastectomy, single-dose esketamine administration has been shown to arrest the decline in postoperative MMSE scores and facilitate cognitive recovery within the first postoperative day (Zhang J. et al., 2023). However, a randomized controlled trial on the effect of esketamine on postoperative delirium in elderly patients undergoing joint arthroplasty showed that esketamine significantly reduced postoperative pain and the use of rescue analgesia, but was associated with higher rates of hallucinations, dizziness, and nightmares. Thus, this suggests that its general applicability may be limited (Zhu et al., 2026). Esketamine, as a viable option for pediatric anesthesia, offers synergistic anti-anxiety and analgesic effects, potentially helping to reduce the incidence of postoperative complications in children and shorten recovery time. For children, esketamine is often administered intranasally, with a bioavailability of 43%–50%. Studies have shown that administering 0.5 mg/kg of intranasal esketamine combined with 1 μg/kg of dexmedetomidine 30–40 min before surgery improves children’s compliance and success rate of sedation during anesthesia induction, while also shortening recovery time. Furthermore, this combination reduces the incidence of pediatric emergence delirium (ED) in the PACU and decreases the severity of postoperative agitation on postoperative day 7 (Pak et al., 2026). However, it should still be noted that esketamine may exert neurotoxic effects on the developing brain. In newborns and early childhood, high-dose or prolonged exposure to ketamine may induce neuronal apoptosis, leading to persistent adverse effects on the structural and functional integrity of the brain. Nevertheless, *in vitro* studies have shown that low concentrations of ketamine can enhance the survival rate of fetal rat hippocampal neurons, inhibit apoptosis, and improve neural plasticity. Currently, most data regarding neurotoxicity come from basic research or short-term clinical observations, with insufficient evidence available on long-term cognitive impacts (Wang J. et al., 2025; Schwenk et al., 2021; Wu et al., 2023).

Similarly, in elderly ischemic stroke patients undergoing neurointerventional procedures, subclinical doses of esketamine (0.25 mg/kg) were associated with improved Mini-Mental State Examination (MMSE) scores and reduced inflammatory markers such as NLRP3 and interleukins, suggesting a neuroprotective effect mediated by anti-inflammatory pathways (Wang L. et al., 2025). A study on the impact of perioperative neurocognitive disorders in elderly patients undergoing gastrointestinal surgery showed that esketamine can reduce inflammatory factors such as IL-6, TNF-α and S100 calcium-binding protein β (S100β), confirming its effectiveness in anti-inflammatory and neuroprotective properties (Lin et al., 2024; Hung et al., 2025).

Overall, despite these promising findings, the majority of clinical studies have relatively small sample sizes (mostly fewer than 200 participants), are often single-center, and lack long-term follow-up data to conclusively establish the safety and sustained efficacy of esketamine for perioperative brain protection. Moreover, heterogeneity in patient populations, surgical procedures, dosing regimens, and outcome measures limits the generalizability of results. Therefore, there is a critical need for large-scale, multicenter RCTs with extended follow-up to validate these preliminary findings and to better define the neuroprotective role of esketamine in diverse surgical settings.

### Optimization of dose and timing of administration

The optimization of esketamine dosing and timing in the perioperative period is essential to maximize neuroprotective benefits while minimizing adverse effects. Current consensus guidelines recommend low-dose intravenous administration, typically between 0.2 and 0.5 mg/kg as a single bolus or short-term continuous infusion at rates of 0.1–0.3 mg/kg/h. This dosing range is considered effective in providing neuroprotection and analgesia without eliciting significant psychotomimetic side effects such as hallucinations or dissociation, which are more prevalent at higher doses (Wang Y. et al., 2024). Studies have found that intravenous administration of esketamine during orthopedic surgery (loading dose: 0.2–0.5 mg/kg; maintenance dose: 0.25 mg/kg/h) effectively alleviates postoperative pain in orthopedic patients, reduces opioid consumption within the first 24 h after surgery, and tends to lower the incidence of chronic pain at 6 months postoperatively (Wang et al., 2026). In procedural sedation and analgesia under emergency conditions, a single intravenous dose of esketamine at 0.125–0.25 mg/kg combined with opioids, or 0.5 mg/kg administered alone, followed by a maintenance infusion of 0.3–1.5 mg/kg/h, typically maintains relatively stable hemodynamic parameters and induces only mild respiratory depression in most critically ill patients (Haske et al., 2023; Trimmel et al., 2018).

Clinically, administering medication during the peak of surgical stress—such as at the time of skin incision or early reperfusion—may enhance the efficacy of esketamine by modulating neuroinflammation and oxidative stress, thereby reducing the incidence of POD and postoperative cognitive dysfunction (Ferreira et al., 2023). However, the precise timing and duration of infusion require further elucidation through rigorously designed clinical trials to establish standardized protocols that balance efficacy and safety.

### Implementation of safety monitoring and individualized management

The perioperative use of esketamine necessitates vigilant safety monitoring and individualized dosing strategies to mitigate potential adverse effects and optimize therapeutic outcomes. Continuous monitoring of cardiovascular parameters such as electrocardiogram (ECG), blood pressure, and heart rate is crucial, given esketamine’s known hemodynamic effects including transient hypertension and tachycardia (Kamp et al., 2021). Sedation depth should be assessed using validated scales like the Richmond Agitation-Sedation Scale (RASS), with dose adjustments or discontinuation considered if sedation exceeds a RASS score of −2 to prevent oversedation and respiratory compromise. Special consideration is warranted in elderly patients, those with hepatic impairment, or individuals receiving concomitant medications that inhibit CYP3A4 enzymes, such as diltiazem, as these factors can significantly alter esketamine metabolism and plasma concentrations, necessitating dose reductions of 40%–50% to avoid toxicity (Nguyen et al., 2022). Emerging evidence supports the development of precision medicine approaches incorporating genetic polymorphisms, particularly CYP2B6 variants, to tailor esketamine dosing and improve safety profiles (Nguyen et al., 2022). Furthermore, integrating real-time monitoring tools such as electroencephalography (EEG) and cerebral oxygen saturation measurements may provide objective assessments of esketamine’s neurophysiological effects, enabling dynamic dose adjustments to optimize neuroprotection. For preoperative assessment of elective surgery patients undergoing anesthesia or sedation, systematic screening for substance use disorders (SUD) must be incorporated. Informed consent should go beyond standard discussions of efficacy and include comprehensive disclosure of known risks, such as cognitive impairment, urinary adverse effects, addiction potential, and drug interactions. Additionally, necessary evaluation and monitoring are required after discharge to address addiction risk warnings and track adverse events. Future research should focus on validating these individualized strategies and establishing comprehensive perioperative protocols to safely implement esketamine in diverse patient populations.

## Conclusion

Growing evidence now indicates that ketamine holds significant clinical value across various medical settings. As an NMDA receptor antagonist, esketamine shares the same pharmacological properties as ketamine, yet exhibits stronger anesthetic, analgesic, and antidepressant effects. Overall, esketamine demonstrates significant potential in perioperative brain protection through direct inhibition of neuronal apoptosis, modulation of neuroinflammation, and reshaping of the gut microbiota. In the future, this field holds promise for developing more effective and personalized perioperative neuroprotective strategies through a multidisciplinary approach integrating neuropsychopharmacology, immunology, microbiology, and clinical science. Continued rigorous research is essential to fully unlock the potential of esketamine, ultimately improving cognitive outcomes and quality of life for surgical patients at risk of neurocognitive complications.
